# The role of *Anopheles arabiensis* and *Anopheles coustani* in indoor and outdoor malaria transmission in Taveta District, Kenya

**DOI:** 10.1186/1756-3305-6-114

**Published:** 2013-04-20

**Authors:** Joseph M Mwangangi, Ephantus J Muturi, Simon M Muriu, Joseph Nzovu, Janet T Midega, Charles Mbogo

**Affiliations:** 1 Kenya Medical Research Institute (KEMRI), Centre for Geographic Medicine Research Coast, P.O. Box 42880108, Kilifi, Kenya; 2 Malaria Public Health Department, KEMRI-Wellcome Trust Research Program, P.O. Box 4364000100, Nairobi, Kenya; 3 Illinois Natural History Survey, University of Illinois, Urban-Champaign, USA; 4 Department of Life Sciences, Imperial College London, South Kensington campus, London SW7 2AZ, UK

## Abstract

**Background:**

The scaling up of malaria vector control efforts in Africa has resulted in changing the malaria vectorial systems across different ecological settings. In view of the ongoing trends in vector population dynamics, abundance, species composition and parasite infectiousness, there is a need to understand vector distribution and their contribution to malaria transmission to facilitate future planning of control strategies. We studied indoor and outdoor malaria transmission dynamics and vector population variability of *Anopheles* mosquitoes in Taveta district along the Kenyan Coast.

**Methods:**

*Anopheles* mosquitoes were collected indoors and outdoors in 4 ecologically different villages using CDC light traps (both indoor and outdoor) and aspiration method (day resting indoors; DRI) methods. Mosquitoes were examined for infection with *P. falciparum* sporozoites and blood feeding preferences using enzyme linked immunosorbent assay (ELISA). The *An. gambiae* and *An. funestus* complexes were identified by PCR technique to determine the sibling species composition.

**Results:**

A total of 4,004 *Anopheles* mosquitoes were collected consisting of 34.9%% (n = 1,397) *An. gambiae* s.1., 28.1% (n = 1,124) *An. funestus* s.l., 33.5% (n = 1,340) *An. coustani* and 3.6% (n = 143) *An. pharoensis*. A total of 14,654 culicine mosquitoes were collected, mainly *Cx. quinquefasciatus*. Of the total *Anopheles* collected, 3,729 were tested for *P. falciparum* sporozoite infection. The sporozoite transmission was found to be occurring both indoors and outdoors. The overall sporozoite infectivity was 0.68% (n = 2,486) indoors and 1.29% (n = 1,243) outdoors. Indoor and outdoor sporozoite infectivity and the vectorial systems varied across the 4 ecological villages. Entomological inoculation rates for the 4 villages indicate that there was site-to-site variation. In the 4 villages, Mwarusa had the highest EIRs with *An. arabiensis, An. funestus* and *An. coustani* contributing to 23.91, 11.96 and 23.91 infectious bites per person per year ib/p/year respectively. In Kiwalwa and Njoro outdoor EIR was significantly higher than indoors.

**Conclusions:**

This study shows that malaria transmission is occurring both indoors and outdoors. The main vectors are *An. arabiensis, An. funestus* and *An. coustani* indoors while *An. coustani* is playing a major role in outdoor transmission. Effective malaria control programmes, should therefore include tools that target both indoor and outdoor transmission.

## Background

The malarial vectorial system in Africa is complex, comprising typically of *Anopheles gambiae*, *An. arabiensis*, and *An. funestus* as the primary vectors and a number of complementally vectors including *An. pharoensis*, *An. coustani* and *An. rivurolum*[1-5]. While the ecology and behavior of the primary vectors is well documented [6-9], little is known about the ecology and behavior of complementally vectors. Previously considered unimportant, the role of these vectors in malaria transmission has increased over the years spurred by the widespread use of insecticide-treated bed nets that are selectively decimating *An. gambiae* and *An. funestus* populations while impacting little on complementally species and one primary vector *An*. *arabiensis*[10-12]. In order to maintain recent gains in malaria control and to proceed towards the intended target of malaria elimination, it will be important to develop strategies to interrupt malaria transmission by these vectors. This effort requires indepth knowledge of the ecology and behavior of these vectors.

To gauge levels of malaria control necessary to achieve meaningful public health improvements in Africa, it will be necessary to quantitatively define the extent to which site-specific entomological inoculation rates (EIRs) must be reduced to correspondingly reduce malaria prevalence [13-15]. A foundation of malaria vector control is that actions to decrease vector-host contact through methods including larval habitat modification, insecticide treatment of larval habitats, spraying insides of houses with residual insecticides, insecticide-treated bed nets, or the use of repellents will have correspondingly beneficial outcomes in terms of reduction in morbidity and mortality. Effective vector control measures decrease the incidence of malaria infections because there is a linear relationship between EIRs and malaria incidence [6,16-18].

In the 1940s–60s, indoor residual spraying (IRS) primarily using dichlorodiphenyltrichloroethane (DDT) reduced the incidence of malaria to zero, or to near zero, in regions where malaria was endemic [19,20]. The effectiveness of DDT against indoor resting mosquitoes led to the view that malaria could be eradicated through a combination of indoor residual spraying (IRS) and disease surveillance to detect and treat any remaining infections. In line with this notion, the Eighth World Health Assembly adopted the concept of malaria eradication resulting in the birth of the Global Eradication Program of Malaria in 1955 [21]. However, by the end of the 1960s it became evident that technical problems, such as resistance of mosquito vectors to insecticides such as DDT, HCH, and dieldrin and resistance of malaria parasites to drugs, presented serious obstacles to the pursuit of eradication programs in many tropical countries [22-25]. In 1969 the World Health Organization recommended that although eradication of malaria should remain an ultimate goal, in countries where eradication does not appear to be feasible, malaria control operations may form a transitional stage [22]. During the second period, from 1965 to 1974, it became clear that the prospects for malaria control (let alone those for eradication) were related to the availability of a network of basic health services [22-25].

During the World Health Organization (WHO) malaria eradication program in 1955, the East African colonies established the Pare-Taveta malaria control scheme, on the Kenya-Tanzania border with its headquarters in Taveta. This program conducted a large-scale trial in the Taveta sub-district of Kenya and the Pare district of Tanzania to determine whether malaria transmission could be interrupted through adoption of indoor residual spraying (IRS) [26,27]. During this expansive malaria control program, entomological and parasitological surveillance systems were used to monitor the changes in human malaria cases and the risk of malaria transmission. The IRS program not only reduced mortality due to malaria by half in all age groups but also eliminated *An. funestus*. In addition, there was a 7-fold reduction in *An. gambiae* populations as well as a reduction in sporozoite rates to undetectable levels [19,20,26]. Despite this success, this program was discontinued in 1960s and as a result, *An. funestus* was reported 6 years later [28] and has recently been reported in this region [29].

In efforts to update the malarial vectorial system of these regions, field surveys were conducted to determine the relative abundance of malaria vectors and their role in malaria transmission in the Taveta area. Results of this research are particularly important in informing the policy makers in planning future interventions especially in agro-ecologic areas where there is a scale up of LLINs distribution.

## Methods

### Study sites

The study was conducted in Taveta, one of the 14 districts in the Coastal province, Kenya. The district lies between latitude 3° 24′00″ S and longitude 37° 41′00″ E. Taveta district is about 109 km West from Voi town off the Nairobi-Mombasa road and is mainly inhabited by the Taveta ethnic group. The occupation of the people in this district is mainly casual waged labor, mixed farming, livestock and trade/business.

The area is a fairly plain terrain that generally slopes towards the south. The area is about 752 m above sea level. Rainfall in the district is inadequate, bimodal and very erratic. The mean annual rainfall ranges between 200 mm and 1,200 mm. The long rains fall between March and May and the short rains occur between November and December. Temperature ranges from 21°C to 31°C. The highest evaporation rate is experienced during the months of January to March. Agricultural activities in this area include horticulture (growing of tomatoes, kales, bananas), livestock farming (cattle, goat, sheep, poultry and bee keeping) and subsistence farming (growing of maize, beans, French beans and sugar) mainly through irrigation. Water for this purpose is derived from four rivers; Tsavo, Lumi, Njoro and Kitobo and spring water from the foot of Mt. Kilimanjaro.

**Figure 1:**
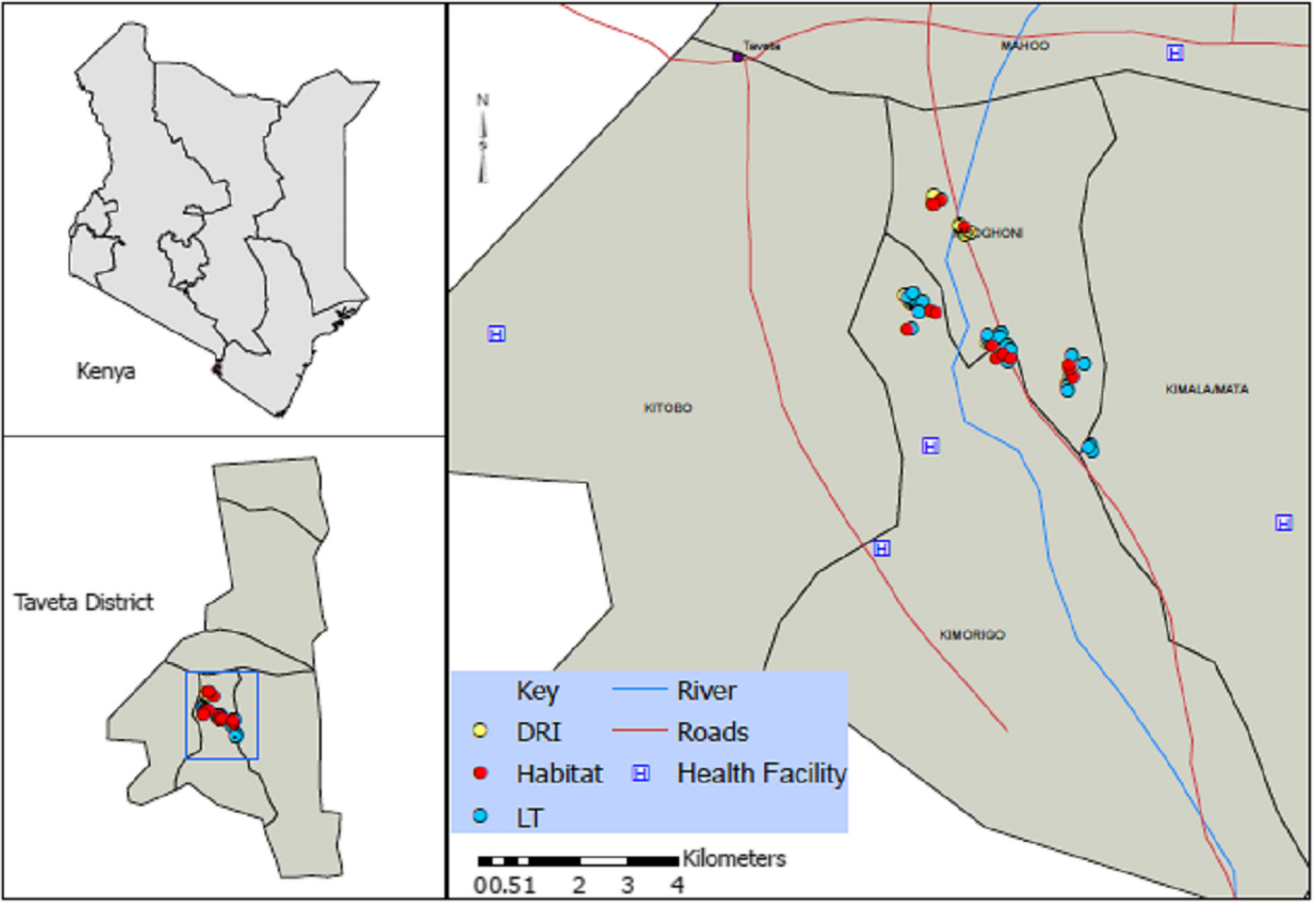
Map showing study site.

Four villages representing distinct ecological zones were sampled for adult mosquitoes. These included Kiwalwa, Mwarusa, Kimundia and Njoro (Figure 1). Kiwalwa is a highly populated riverine ecosystem with clustered houses. Mwarusa village is a fairly flat and swampy area and river Lumi flows along the edge of the village. The households are sparsely distributed and the homesteads consist of three or more houses. Kimundia village is swampy and households are sparsely distributed and the homesteads consist of one or two houses. During the rainy season, some sections of this village are flooded and inaccessible. Njoro village is mainly in a semi-arid ecosystem and is typically dry and dusty during the dry season. The coordinates of mosquito collection stations in each village were taken using hand held GPS machines (Garmin International Inc., Olathe, KS) and used to develop base maps (ArcGIS 10).

The entomological sampling design was based on rainfall pattern and the strategy covered the long wet season (March and May), long dry season (June to October), short wet season (November and December) and short dry season (January to March). A comprehensive entomological sampling was done for two weeks in every season in 2011.

### Mosquito collection

Mosquitoes were collected using standard Centre for Disease Control light traps (CDC, Atlanta, GA, USA) and manual aspiration techniques [30]. For each village, the collections were conducted in 10 randomly selected houses between 1800 and 0600 hrs. The selection criteria included the location and type of house (center, periphery, mud-walled, grass-thatched), presence or absence of aquatic habitats, and accessibility. One light trap was hung from the ceiling at the foot end of the bed and a second trap was positioned outdoors in the same house. The traps were removed in the morning and all cups containing mosquitoes were kept in the cool box for transportation to the laboratory for further processing.

Ten different houses were selected in each village for manual mouth aspiration of indoor resting mosquitoes between 0600 and 1000 hrs and transported to the laboratory for further processing.

### Mosquito identification and processing

The mosquitoes were sorted to species and sex using taxonomic keys [31,32]. Female *Anopheles* mosquitoes were cut transversely between the thorax and abdomen. The head and thorax region was used for testing the sporozoite infectivity using *Plasmodium falciparum* sporozoite enzyme linked immunosorbent assay (ELISA) [33-35]. Blood-engorged females were also tested for blood meal sources by ELISA [36].

The wings and legs for *An. gambiae* complex were preserved in silica gel and further identified to sibling species by rDNA Polymerase Chain Reaction (PCR) [37,38]. Genomic DNA was extracted from the legs and wings of a proportion of females in the two complexes using the methods of Collins *et al.*[39,40] and amplified using specific diagnostic primers for *An. gambiae s.s., An. arabiensis,* and *An. merus* for *An. gambiae* complex [38,41].

### Data analysis

Data was examined for normality and homogeneity of variance using Kolmogorov-Smirnov and Levene tests, respectively and log transformation was conducted to correct for deviation from normality. Repeated measures ANOVA with Greenhouse-Geisser correction was used to determine the effect of time, species, collection method and site on the mean number of adult mosquitoes collected.

#### Entomologic indices

*Plasmodium falciparum* sporozoite rates were calculated by expressing the number of *P. falciparum* positive mosquitoes as a percent of the total number of mosquitoes tested per species. The feeding preference was calculated by expressing the number of mosquitoes positive for each specific host as a proportion of the total mosquitoes tested. Human Blood Index (HBI) was calculated as the proportion of the mosquitoes positive for human blood divided by number tested successfully for blood meal analysis. The malaria transmission indices were determined by calculating the entomological inoculation rates (EIR). EIR was obtained for the day resting indoor aspiration by multiplying the human-biting rate by the proportion of sporozoite positive mosquitoes. The human-biting rates (the number of biting mosquitoes per human-night), was derived by dividing the total number of blood-fed and half-gravid mosquitoes caught by the number of persons sleeping in the house the night preceding the collection and multiplying the resulting value with HBI [6]. For the light trap collections, EIR was estimated including a conversion factor for Light trap catches vs. man biting catches of 1.605, as described earlier [42,43], without allowance for the number of occupants per room. The overall annual EIR for the LT, was calculated using standard methods, i.e. 1.605 × (*no*. *of sporozoite positive ELISA*/*no*. *of mosquitoes tested*)/(*no*. *of mosquitoes collected*/*no*. *of catches*) × 365.

### Ethical considerations

Verbal consent was obtained from the household head or their representative before commencing mosquito collection. These mosquito surveys were performed under human investigations protocols approved by the Ethical Review Board of Kenya Medical Research Institute (Nairobi, Kenya).

## Results

### Mosquito species composition and abundance

A total of 18,658 adult mosquitoes consisting of 1,397 *An. gambiae s.l.*, 1,124 *An. funestus*, 1340 *An. coustani*, 143 *An. pharoensis*, and 14,654 *Cx. quinquefasciatus* were collected using the three techniques (Table 1). The relative abundance of the five mosquito species varied among study sites and collection methods (Table 1). In all the four study sites, *Cx. quiquefasciatus* was the most abundant species in light trap collections both indoors and outdoors. The second most abundant species collected in light traps also varied by trap location and site. In Mwarusa and Njoro, respectively, *An. gambiae s.l.* and *An. funestus* were the second most dominant species in both indoor and outdoor light trap collections. In Kimundia, *An. coustani* was the second most dominant species in outdoor light trap collections while *An. gambiae* s.l. was the second most dominant species in indoor light trap collections. In Kiwalwa, *An. coustani* was the second most dominant species in outdoor light traps while *An. gambiae s.l.* and *An. constani* were equally the second dominant species in indoor light traps (Table 1). In DRI collections, *An. gambiae s.l.* was the most abundant species in all study sites except Kiwalwa where *An. funestus* was the most dominant species (Table 1). In some sites but not others, *An. gambiae* s.l. and *An. funestus* were collected indoors rather than outdoors (Table 1). Conversely, there was a trend for collecting *An. coustani* more outdoors than indoors (Table 1).

**Table 1:** The number of mosquitoes collected and their relative abundance from each village

**Village**	**Method**	***An. gambiae*s.l.**	***An. funestus***	***An. coustani***	***An. pharoensis***	***Cx. quinquefasciatus***
Kimundia	DRI	202 (50.1)*	29 (7.2)	9 (2.2)	0 (0.0)	163 (40.4)
	LT Indoor	187 (6.3)	48 (1.6)	62 (2.1)	65 (2.2)	2,587 (87.7)
	LT Outdoor	20 (0.9)	36 (1.6)	346 (15.4)	9 (0.4)	1,836 (81.7)
	**Sub-total**	**409 (7.3)**	**113 (2.0)**	**417 (7.4)**	**74 (1.3)**	**4,586 (81.9)**
Kiwalwa	DRI	153 (30.7)	200 (40.1)	0 (0.0)	2 (0.0)	144 (28.9)
	LT Indoor	235 (14.8)	366 (23.2)	231 (14.5)	22 (1.4)	735 (46.3)
	LT Outdoor	76 (1.0)	130 (1.7)	526 (7.0)	7 (0.1)	6,800 (90.2)
	**Sub-total**	**464 (4.8)**	**696 (7.2)**	**757 (7.9)**	**31 (0.3)**	**7,679 (79.8)**
Mwarusa	DRI	169 (54.7)	33 (10.7)	2 (0.6)	1 (0.3)	104 (33.7)
	LT Indoor	185 (30.1)	71 (11.5)	50 (8.1)	8 (1.3)	301 (48.9)
	LT Outdoor	60 (4.5)	26 (1.9)	59 (4.4)	24 (1.8)	1,165 (87.3)
	**Sub-total**	**414 (18.3)**	**130 (5.8)**	**111 (4.9)**	**33 (1.5)**	**1,570 (69.5)**
Njoro	DRI	73 (43.7)	58 (34.7)	0 (0.0)	1 (0.6)	35 (21.0)
	LT Indoor	18 (5.3)	50 (14.7)	15 (4.4)	2 (0.6)	254 (74.9)
	LT Outdoor	19 (2.8)	77 (11.5)	40 (6.0)	2 (0.3)	530 (79.3)
	**Sub-total**	**110 (9.4)**	**185 (15.8)**	**55 (4.7)**	**5 (0.4)**	**819 (69.8)**

*Parenthesis shows percentage.

Of the 459 *An. gambiae* s.l. samples that were identified to sibling species by PCR, 97.60% were characterized as *An. arabiensis* and the rest were *An. gambiae s.s* (2.40%) (n = 11)*.*

### Mosquito density

The mean number of *Cx. quinquefasciatus* was 117.5 ± 43.3 (mean ± SE) and was significantly higher than 10.78 ± 1.3, 8.1 ±1.0, 7.2 ± 2.0, and 3.0 ± 0.8 for *An. arabiensis*, *An. funestus*, *An. coustani* and *An. phoroensis*, respectively (F = 6.5, df = 4, 603, P < 0.001) (Table 2). The mean number of *An. pharoensis* was also significantly lower than those of *An. arabiensis* and *An. funestus*. The mean number of adults collected varied by site, species, and collection method (F = 3.548, df = 8,769, 550.251). Regardless of the type of trap, Njoro had significantly lower numbers of *An. arabiensis* compared to the other sites. Similarly for all trap types, Kiwalwa had a significantly higher number of *An. funestus* compared to the other villages. In addition, the mean number of *An. funestus* collected with DRI and LT indoors was lowest in Kimundia, whereas for LT outdoors, the numbers were significantly lower in Mwarusa compared to the other villages. For *An. coustani* the mean number of adults collected by DRI was significantly higher in Kimundia and Mwarusa compared to Kiwalwa and Njoro, whereas for LT indoors and outdoors, significantly higher numbers were collected in Kiwalwa compared to the other villages. Finally, the mean number of *An. pharoensis* collected in both LT indoors and LT outdoor traps was significantly higher in Kimundia compared to the other villages.

**Table 2:** Mean number of adult mosquitoes collected in the five villages using different collection methods

		**Village**			
**Method**	**Species**	**Kimundia**	**Kiwalwa**	**Mwarusa**	**Njoro**
DRI	*An. arabiensis*	17.4 ± 6.1	13.9 ± 3.1	16.9 ± 4.3	6.7 ± 2.8
	*An. funestus*	1.3 ± 0.6	13.2 ± 7.2	3.0 ± 1.5	5.6 ± 1.0
	*An. coustani*	0.8 ± 0.6	0.0 ± 0.0	0.8 ± 0.2	0.0 ± 0.0
	*An. pharoensis*	0.0 ± 0.0	0.0 ± 0.0	0.0 ± 0.0	0.1 ± 0.1
	*Cx. quinquefasciatus*	16.4 ± 4.6	13.1 ± 2.7	4.7 ± 1.7	4.0 ± 1.5
LT indoor	*An. arabiensis*	18.7 ± 8.1	24.3 ± 5.9	18.1 ± 6.1	2.1 ± 0.8
	*An. funestus*	3.0 ± 1.1	37.1 ± 2.9	8.1 ± 0.2	5.0 ± 2.1
	*An. coustani*	6.2 ± 1.7	13.9 ± 4.5	4.0 ± 1.3	1.5 ± 1.0
	*An. pharoensis*	6.5 ± 3.2	1.6 ± 1.1	0.8 ± 0.5	0.2 ± 0.1
	*Cx. quinquefasciatus*	255.7 ± 101.5	75.5 ± 15.8	30.9 ± 5.7	24.5 ± 5.3
LT outdoor	*An. arabiensis*	2.8 ± 1.0	6.8 ± 1.9	3.0 ± 2.4	1.2 ± 0.6
	*An. funestus*	3.8 ± 1.1	13.8 ± 2.9	0.6 ± 0.2	7.2 ± 2.1
	*An. coustani*	5.1 ± 2.3	43.7 ± 16.8	2.5 ± 1.1	3.4 ± 1.4
	*An. pharoensis*	26.6 ± 5.2	0.6 ± 0.3	0.6 ± 0.4	0.0 ± 0.0
	*Cx. quinquefasciatus*	791.8 ± 477.6	70.8 ± 15.6	84.7 ± 40.8	49.2 ± 23.6

### The *Plasmodium falciparum* sporozoite rates and entomological inoculation rates in malaria vectors in Taveta District

A total of 3,729 *Anopheles* mosquitoes were tested for *P. falciparum* sporozoite infection (Table 3). The sporozoite transmission was found occurr both indoors and outdoors. The overall indoor sporozoite infectivity rate was 0.68% (n = 2,486) for mosquitoes collected indoors and 1.29% (n = 1,243) for mosquitoes collected outdoors. The sporozoite infectivity rate was 0.66% (n = 909) for mosquitoes collected using the aspiration method (Day Resting Indoors), 0.70% (n = 1,577) for mosquitoes collected using Light traps indoors and 1.29% for mosquitoes collected through Light trap outdoors. In the indoor sporozoite infectivity, Mwarusa had the highest sporozoite rates and 3 species namely *An. arabiensis*, *An. funestus* and *An. coustani* were responsible for the transmission. In Kimundia, only *An. arabiensis* was transmitting sporozoites while it was only *An. funestus* transmitting sporozite in Njoro. Sporozoite infected *An. coustani* were collected indoors in Kiwalwa and Mwarusa.

**Table 3:** ***Plasmodium falciparum *sporozoite transmission and Entomological Innoculation Rates (EIR) foci for Taveta District**

			**Village**				
**Index**	**Method**	**Species**	**Kimundia**	**Kiwalwa**	**Mwarusa**	**Njoro**	**Total**
**Sporozoite rates**	DRI	*An. coustani*	14	0	2	0	16
		*An. funestus*	29	200	33	46	308
		*An. arabiensis*	202	139 (0.72%)	167 (2.99%)	73	581 (1.03%)
		*An. pharoensis*	0	2	1	1	4
		**Total**	**245**	**341**	**203 (2.46%)**	**120**	**909 (0.66%)**
	LT Indoors	*An. coustani*	67	231 (0.43%)	50 (4.00%)	3	351 (0.85%)
		*An. funestus*	48	366	71 (1.41%)	50 (2.00%)	535 (0.37%)
		*An. arabiensis*	167 (1.80%)	235 (0.43%)	185 (1.08%)	18	605 (0.99%)
		*An. pharoensis*	56	22	8	0	86
		**Total**	**338 (0.89%)**	**854 (0.23%)**	**314 (1.59%)**	**70 (1.43%)**	**1,576 (0.70%)**
	LT Outdoors	*An. coustani*	346 (0.29%)	456 (2.41%)	59 (1.69%)	40 (7.5%)	901 (1.78%)
		*An. funestus*	36	76	11	77	200
		*An. arabiensis*	15	76	14	19	124
		*An. pharoensis*	9	7	0	2	18
		**Total**	**406 (0.25%)**	**615 (1.79%)**	**84 (1.19%)**	**138 (2.17%)**	**1,243 (1.29%)**
**Annual EIR**	DRI	*An. arabiensis*	0.00	1.14	4.74	0.00	1.64
	LT Indoors	*An. coustani*	0.00	11.72	23.91	0.00	8.49
		*An. funestus*	0.00	0.00	11.96	11.05	5.66
		*An. arabiensis*	31.95	11.72	23.91	0.00	16.98
		**Total**	**31.95**	**23.43**	**59.78**	**11.05**	**31.13**
	LT outdoors	*An. coustani*	17.23	123.92	14.65	45.06	56.81

For the outdoor transmission, *An. coustani* was the main vector and was found to be playing a key role in malaria transmission in the 4 villages. There was more outdoor sporozoite transmission in Njoro and in Kiwalwa. One way ANOVA showed that there was a significant difference in the sporozoite infectivity in the 4 villages (F_(1,3)_, = 3.11, P = 0.025). None of the *An. pharoensis* was found to be positive for sporozoites both indoors and outdoors.

Entomological inoculation rates for the 4 villages showed that there was site-to-site variation. In the 4 villages, Mwarusa had the highest EIRs and 3 vectors were mainly transmitting, which were *An. arabiensis, An. funestus* and *An. coustani* contributing to an estimated 23.91, 11.96 and 23.91 ib/p/year respectively. In Kiwalwa and Njoro outdoor EIR was significantly higher than indoors. *An. arabiensis* was the key vector contributing to the highest annual EIR (16.98 ib/p/year for LT traps) and 1.64 ib/p/year for DRI. Overall EIR for indoors using Light traps was 31.13 infective bites per person per year (ib/p/year) while using the aspiration method (DRI) this was was 1.05 ib/p/year. For the outdoor transmission, EIR was 56.81 ib/p/year.

## Discussion

Our results show that indoor malaria transmission is mainly perpetuated by *An. arabiensis, An. funestus* and *An. Coustani,* while outdoor transmission is sustained by *An. coustani*. Traditionally, malaria transmission in much of Africa has been dominated by *An. gambiae* and *An. Funestus,* which primarily feed and rest indoors where they can be efficiently targeted with domestic insecticides [44-46]. There is growing evidence from across the continent that the widespread use of LLINs and IRS is driving vector species composition toward those with more flexible feeding and resting behaviors [10,47,48]. *Anopheles coustani* has been collected in several parts of Africa but very rarely has been found infected with *P. falciparum*[1,49]. Malaria control experts have undoubtedly continued to deliver interventions that tackle indoor transmission in Africa. However, our results support previous findings that considerable investment in methods that target mosquito populations outdoors is urgently needed to sustain existing levels of malaria control and to make further inroads towards malaria elimination [48,50,51]. Currently in Africa, there is no intervention that specifically targets outdoor biting mosquitoes; instead most malaria vector control policies in Africa are based on the use of LLINs, prompt diagnosis and treatments and malaria in pregnancy [52]. Scale up of LLINs to universal coverage coupled with larval habitat management strategies, stakeholder involvement and community engagement packaged in integrated vector management (IVM) strategies would be ideal to significantly reduce indoor and outdoor resting vectors [53-55]. The only currently operational tool that could provide additive benefit to LLINs is larviciding [56-59], which by killing larval mosquitoes in their aquatic habitats may be assumed to efficiently target both the endophilic and exophilic proportion of vector populations.

Five mosquito species were collected in the 4 villages namely, *An. arabiensis, An. funestus, An. coustani, An. pharoensis* and *Cx. quinquefasciatus*. This implies that the inhabitants of the 4 villages in Taveta district are exposed to both infectious and nuisance biting. There was significant variation in the densities of mosquitoes by site, species, and collection method. Villages that were mainly carrying out agricultural activities, such as Kiwalwa and Kimundia had a significantly higher number of mosquitoes, while arid and semi-arid areas such as Njoro had fewer mosquitoes throughout the year. This shows clearly that mosquito production is a function of the availability of larval habitats [60,61], with more mosquitoes being found in areas with available water for agricultural activities.

In Kiwalwa and Njoro, outdoor entomological inoculation rate (EIR) was higher than indoor EIR. This result shows that the level of exposure to *P. falciparum*-infected mosquitoes is higher outdoors as compared to indoors. EIR assessments in these villages show that it is important while conducting malaria transmission studies to consider estimating in both indoor and outdoor environments. This is because as efforts are increased to reduce human–vector contact indoors, this may have an effect in increasing outdoor transmission. Further, estimation of EIR using CDC light traps was found to be better compared to using a manual aspiration technique, which captured indoor day resting mosquitoes. However, there is a need to come up with more studies to evaluate the existing mosquito sampling tools in the view of current vectorial shifts in behavior and in composition [10,12,62]. In this study CDC light traps were used for outdoor and indoor sampling since some earlier studies have shown that CDC light traps are effective for indoor collection of host seeking mosquitoes, with a catch that compares well with the standard human landing catch method [42,43]. This study has shown that there is outdoor transmission of malaria, there is need to enhance outdoor sampling and more sampling tools need to be evaluated to ensure outdoor collections are optimized.

In this study, CSP-ELISA, which is widely used to estimate the sporozoite index, was used to gauge the level of malaria transmission [6,63-69]. However, several studies have reported false positive results using CSP-ELISA [70-74]. To overcome some of challenges posed by CSP-ELISA, in particular, false positivity, it would be advantageous if future malaria transmission studies could be designed to detect sporozoites in mosquitoes using *Plasmodium* specific polymerase chain reaction (PCR) [74]. PCR should be able to detect very few sporozoites in a sample compared to ELISA, which requires several sporozoites.

## Conclusion

In conclusion, this study shows that malaria transmission is occurring both indoors and outdoors. Indoor transmission is mainly due to *An. arabiensis, An. funestus* and *An. coustani* inside houses while *An. coustani* is playing a major role in outdoor transmission. For effective malaria control programs, efforts should be made to incorporate more tools that target both indoor and outdoor transmission.

## Competing interest

The authors declare that they have no competing interest.

## Authors’ contributions

JMM, EJM, SMM, JN and JTM conducted all the field and laboratory work. CMM provided scientific guidance in data collection, analysis and manuscript preparation and planning, and implementation of day-to-day field activities and offered scientific guidance in data analysis and manuscript preparation. All authors actively contributed to the interpretation of the findings and development of the final manuscript and approved the final manuscript.
